# Solvent Evaporation Rate as a Tool for Tuning the Performance of a Solid Polymer Electrolyte Gas Sensor

**DOI:** 10.3390/polym14214758

**Published:** 2022-11-06

**Authors:** Petr Sedlak, Pavel Kaspar, Dinara Sobola, Adam Gajdos, Jiri Majzner, Vlasta Sedlakova, Petr Kubersky

**Affiliations:** 1 Department of Physics, Faculty of Electrical Engineering and Communications, Brno University of Technology, Technicka 10, 616 00 Brno, Czech Republic; 2 Research and Innovation Centre for Electrical Engineering (RICE), Faculty of Electrical Engineering, University of West Bohemia, Univerzitni 8, 301 00 Plzen, Czech Republic

**Keywords:** solid polymer electrolyte, gas sensor, noise spectroscopy, ionic liquid

## Abstract

Solid polymer electrolytes show their potential to partially replace conventional electrolytes in electrochemical devices. The solvent evaporation rate represents one of many options for modifying the electrode–electrolyte interface by affecting the structural and electrical properties of polymer electrolytes used in batteries. This paper evaluates the effect of solvent evaporation during the preparation of solid polymer electrolytes on the overall performance of an amperometric gas sensor. A mixture of the polymer host, solvent and an ionic liquid was thermally treated under different evaporation rates to prepare four polymer electrolytes. A carbon nanotube-based working electrode deposited by spray-coating the polymer electrolyte layer allowed the preparation of the electrode–electrolyte interface with different morphologies, which were then investigated using scanning electron microscopy and Raman spectroscopy. All prepared sensors were exposed to nitrogen dioxide concentration of 0–10 ppm, and the current responses and their fluctuations were analyzed. Electrochemical impedance spectroscopy was used to describe the sensor with an equivalent electric circuit. Experimental results showed that a higher solvent evaporation rate leads to lower sensor sensitivity, affects associated parameters (such as the detection/quantification limit) and increases the limit of the maximum current flowing through the sensor, while the other properties (hysteresis, repeatability, response time, recovery time) change insignificantly.

## 1. Introduction

The rise of solid polymer electrolytes (SPEs) has given the world a material with a wider use than the standard liquid electrolyte, for use in the field of energy storage, such as in capacitors, fuel cells or batteries, sensors, or in other electrochemical devices [1,2,3,4,5,6]. Solid polymer electrolytes show potential for fulfilling this demand, given their good electrochemical stability and mechanical properties. Additionally, SPE-based electrochemical gas sensors can operate at ambient temperature, preventing some problems associated with the instability of the parameters of gas sensors working at high temperatures [7]. SPEs can take many forms, but they are all basically ion-conducting membranes consisting of a salt dispersed in a polymer matrix forming an ionically conducting solid solution [8,9]. There are many variants [1,4,8,10,11,12,13,14], but one of the more prominent choices for SPE is a room temperature ionic liquid (IL) blended (immobilized) in a polyvinylidene fluoride (PVDF) host, which is interesting due to its chemical, thermal and electrical stability [5] and has a wide range of applications [15,16]. These ILs have different properties from ordinary molecular liquids, namely high viscosity and low vapor pressure, due to the strong electrostatic interactions between the oppositely charged ions [17]. The basic properties of ILs have been the target of much research and are well documented [18,19,20,21]. These properties, including good thermal and chemical stability and non-flammability [22], and the easy incorporation of ILs into the form of a solid polymer electrolyte, make them an excellent candidate as a conductive component for SPEs. There are, naturally, downsides to using ILs as electrolytes when compared to conventional electrolyte solutions, specifically their significantly lower conductivity. That said, the ionic conductivity of ILs is sufficiently high, and the other excellent properties of this type of material, including their large electrochemical window and modifiability [23], together with their stability, meaning they are more than suitable for sensor preparation. Sensors with SPEs were used to create a NO_2_ sensor [24,25,26,27,28], sulfur dioxide sensor [29], methane sensor [30] and an ethylene sensor [23]. SPEs continue to prove versatile and valuable for use in sensors, and the work presented here aims to add to the pool of sensor variants and applications. Several authors report changes to the electromechanical properties of PVDF/IL electrolytes caused by thermal treatment. Thermal annealing could lead to an increase in the polar phase and a decrease in the degree of crystallization of PVDF composites, as well as an increase in ion mobility induced by the segmental motion of the polymer chains and polar phase crystals [31,32,33]. Other researchers show that the solvent evaporation temperature significantly influences the morphology, degree of crystallinity, mechanical properties and electrical conductivity [9,34,35,36,37].

The selection of the ionic liquid for the SPE is important for the performance of the electrochemical sensors as well as for their technological preparation. The sensitivity of an amperometric sensor depends on the electrical conductance of the resulting IL-based SPE [25,38] and also on the microstructure of the IL-based SPE [24,25,39], while the effect of solvent evaporation on sensor sensitivity was slightly indicated in previous studies [24,25]. These facts raise the question of how solvent evaporation affects the overall performance of an SPE-based amperometric sensor.

The main focus of this paper is to deliver the answer to this question. This work follows from previous research that described the effect of thermal treatment on the morphology of PVDF/IL-based solid polymer electrolytes [37], AC/DC conductivity and also current fluctuations of PVDF/IL-based solid polymer electrolyte [9] as well as the preparation, electrical and noise characterization of amperometric sensors [40,41,42,43]. The paper describes the effect of solvent evaporation on sensor parameters, such as sensitivity, limit-of-detection, saturation current, signal-to-noise ratio, etc. The paper also describes an equivalent electric circuit of the presented sensor and the morphology of the WE–SPE interfaces where the SPE microstructure is bound to the particular crystallization pathways during the preparation of the electrolyte. 

## 2. Materials and Methods

### 2.1. Sensor Preparation

**Figure 1:**
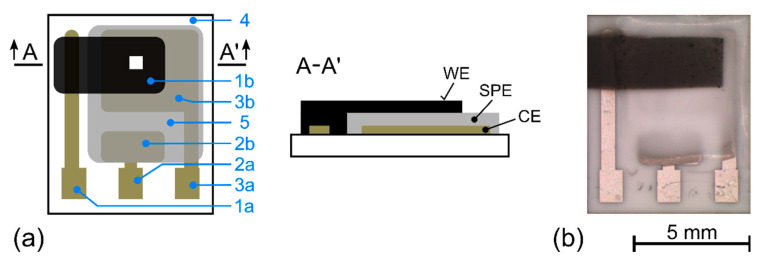
Topology of an amperometric NO_2_ sensor (**a**): working electrode contact (1a), carbon working electrode (1b), pseudo-reference electrode contact (2a), pseudo-reference electrode (2b), counter electrode contact (3a), counter electrode (3b), substrate (4), solid polymer electrolyte (SPE) (5), white square indicates the location of Raman spectroscopy measurements of the WE electrode. (**b**) Sensor with MWCNT working electrode and SPE layer treatment 120 °C for 90 s.

All experimental measurements were performed on a three-electrode sensor platform (Figure 1a) composed of a ceramic substrate with a platinum counter (CE) and a pseudo-reference electrode (RE), a layer of SPE and a carbon-based working electrode (WE). This platform was customized by TESLA Blatná Company (Blatná, Czech Republic) and thoroughly tested and described in previous studies [23,24,38,43,44,45]. A solid polymer electrolyte (SPE) was deposited on the sensor substrate by drop-casting [44] and a glass rod was used to spread the SPE mixture over the required area. A hot plate was then used for thermal treatment of the SPE layers at specific temperatures for specific times in order to obtain the desired variety of porosity and microstructures in the SPE layers. Subsequently, a working electrode based on multiwall carbon nanotubes (MWCNT) was deposited by spray coating on the SPE layer (Figure 1b).

The solid polymer electrode has three main components: (i) ionic liquid 1-ethyl-3-methylimidazolium bis(trifluoromethylsulfony)imide ([EMIM][TFSI], Merck, Darmstadt, Germany), (ii) polymer matrix poly-(vinylidene fluoride) (PVDF, Sigma-Aldrich, St. Louis, MO, USA) and (iii) N-methyl-pyrrolidone (NMP, VWR, Randor, Pennsylvania, USA) as a solvent. The preparation and thermal treatment of the SPE layers were performed under the following conditions: 80 °C for 90 s, 120 °C for 90 s, 120 °C for 210 s and 150 °C for 600 s to achieve different solvent evaporation rates. These conditions were chosen to introduce the largest variability in the morphology and structures of the resulting SPE layers and to achieve the best possible adhesion between the ceramic substrate and the SPE layer [37]. For the preparation of the working electrode, MWCNTs with a length of 10–35 µm and outside diameter of <8 nm (Nanografi, Ankara, Turkey) were prepared in dispersion in ethanol to be deposited by spray coating: 1 mg MWCNT in 3 mL EtOH. The mixture was sonicated in an ultrasonic bath for 24 h to ensure proper dispersion. A polymeric rectangular stencil was used to specify the WE geometrical area of approximately 9 mm^2^ for all the samples. 

### 2.2. Characterization

Scanning electron microscopy (SEM) was performed using a LYRA3 (TESCAN, Brno, Czech Republic) microscope with an acceleration voltage of 10 kV.

Raman spectroscopy, a non-destructive spectroscopic technique, was used to obtain information about the molecular structure and the local chemical environments, as well as the subtle changes in the electrolytes [9,37]. The Raman spectra were collected by a confocal Raman imaging system Alpha 300 R (WITec, Ulm, Germany) with a 50 mW excitation laser at a wavelength of 532 nm, 50× objective magnification and integration time of 2 s.

Electrochemical impedance spectroscopy was measured by a potentiostat/galvanostat (Autolab PGSTAT204, Metroohm AG, Herisau, Switzerland) equipped with a FRA32M module. Measurements were carried out under standard conditions (298 K, 40%RH, 1013.25 hPa, analyte flow rate 1 L/min, V_BIAS_ = −0.5 V vs. Pt pseudo-reference electrode) in the test chamber. A sinusoidal perturbation signal with an amplitude of 5 mV was used within a frequency range from 10 kHz to 5 mHz.

### 2.3. Experimental Setup 

The testing setup comprised mass flow controllers directed by PC, gas cylinders providing synthetic air, a reference calibration mixture (100 ppm NO_2_ balanced in nitrogen), a test chamber, and readout electronics. A more detailed description of the whole setup can be found in previous papers [41,42]. The operational bias voltage was set to −0.5 V vs. platinum RE for all the tested sensors, which were placed on the bottom of the test chamber with gold spring probe contacts. The samples were exposed to various concentrations of nitrogen dioxide ranging from 0 up to 10 ppm under the following conditions: 298 K, 40%RH, 1013.25 hPa, analyte flow rate 1 L/min. Unless otherwise stated, each sensor sample was placed in the same position in the test chamber under the same fluidic conditions. 

Sensor performance was investigated in two ways. We focused on steady-state measurements to analyze the mean value of the current response and its fluctuations using our very sensitive laboratory apparatus operating at higher sampling rates. As this is a very time-consuming procedure, the measurement was only performed for one sample for each type of SPE. The second standard approach simultaneously measures six sensor samples with a commercial potentiostat circuit (LMP91000, Texas Instruments, Dallas, TX, USA). This circuit can be used for a wide range of electrochemical sensing applications, and it was used here to obtain a general overview of the effect of the SPE type on the sensor parameters.

#### 2.3.1. Measurement of Steady-State and Fluctuations

The measurement was performed as follows: firstly, a particular NO_2_ concentration (e.g., 1 ppm) with a total flow rate of 1 L/min (298 K, 40%RH) was set. After a few minutes, when the sensor current response had achieved a steady state value corresponding to the particular NO_2_ concentration level, simultaneous measurements of both direct current and noise were carried out by a two-channel device. The same procedure was then applied to other NO_2_ concentration levels.

Every sensor was a part of a potentiostat circuit based on a rail-to-rail operational amplifier (OPA2144, Texas Instruments, Dallas, TX, USA) with the WE grounded configuration [41,44,46]. The proper noise measurement process is highly dependent on using apparatus and a measurement circuit suitable to the task. Thus, the potentiostat circuit and a low noise transimpedance amplifier were combined to form a single device powered by a battery, providing simultaneous current fluctuation and direct current measurement. The AC voltage output was connected to an amplifier equipped with highly selective filters (AM22, 3S Sedlak, s.r.o., Brno, Czech Republic). It was acquired by a 12-bit AD convertor (HS3, TiePie engineering, Sneek, The Netherlands), which also acquired the DC voltage output as the second channel. To minimize the influence of any power peaks and electric field disturbances, the test chamber with the sensor and our potentiostat-circuit device was placed in a Faraday cage, and all of the devices, including the laptop, were powered by batteries.

#### 2.3.2. Standard Measurement of Sensor Response

A group of six sensors was prepared for each SPE type. These sensors were tested simultaneously in the test chamber using a customized evaluation board equipped with six configurable LMP91000 potentiostats and ΔΣ AD converters (LTC2485, Linear technology, Milpitas, California, USA). All the sensors were tested under identical conditions (298 K, 40%RH, 1013.25 hPa, analyte flow rate 1 L/min), and the signal from each sensor was recorded every other second.

## 3. Results and Discussion

The working electrode–electrolyte interface plays a key role in any amperometric gas sensor because of the chemical reaction occurring on the working electrode where the analyte is either reduced or oxidized. Such a reaction (together with the corresponding half-reaction on the counter electrode) allows electronic conduction in external conductors and electrodes to be coupled to ionic conduction in the electrolyte of an electrochemical cell (sensor). In other words, corresponding half-reactions occurring on the WE and CE electrodes allow the flow of charge carriers through the sensor. This electric current can be used as a signal to quantify the concentration of the detected matter. In this study, the traditional liquid-based electrolyte is replaced by the SPE, and so the electrochemically active area, i.e., the WE–SPE interface, becomes more complicated and depends on more variables. 

Several authors [9,34,35,37,47] have shown that solvent evaporation affects the crystalline phase of PVDF in the solvent. This has implications for the morphology, degree of crystallinity, mechanical properties and ionic conductivity of PVDF-based polymer electrolytes and solvents. 

In the case of our blend of the PVDF matrix, NMP solvent, and ionic liquid [EMIM][TFSI], the thermogravimetric analysis showed that the thermal stability of PVDF and IL is higher (higher boiling point) than NMP solvent [17]. This is characterized by two well-separable processes (at 110 °C and 425 °C) in the SPE mixture. The first process is solvent evaporation, which affects the morphology and conductivity of the SPE. Our thermogravimetric analysis [37] then estimated the mass loss of the solvent (NMP) from our SPE mixture: 5.5%, 32%, 58% and 100% for the following conditions for thermal treatment (a) 80 °C 90 s, (b) 120 °C 90 s, (c) 120 °C 210 s and (d) 160 °C 600 s, respectively. Neither IL nor PVDF were found to evaporate from the SPE mixture under these conditions. 

Below, the authors discuss the following issues: (i) morphology of the WE–SPE interface revealed by scanning electron microscopy (SEM) and Raman spectroscopy to describe MWCNT-based WE and different SPE microstructures for various thermal treatment conditions, (ii) description of the sensor by an equivalent electric circuit, (iii) effect of solvent rate evaporation on sensor performance.

### 3.1. Morphology of the Working Electrode/Solid Polymer Electrolyte (WE–SPE) Interface

**Figure 2:**
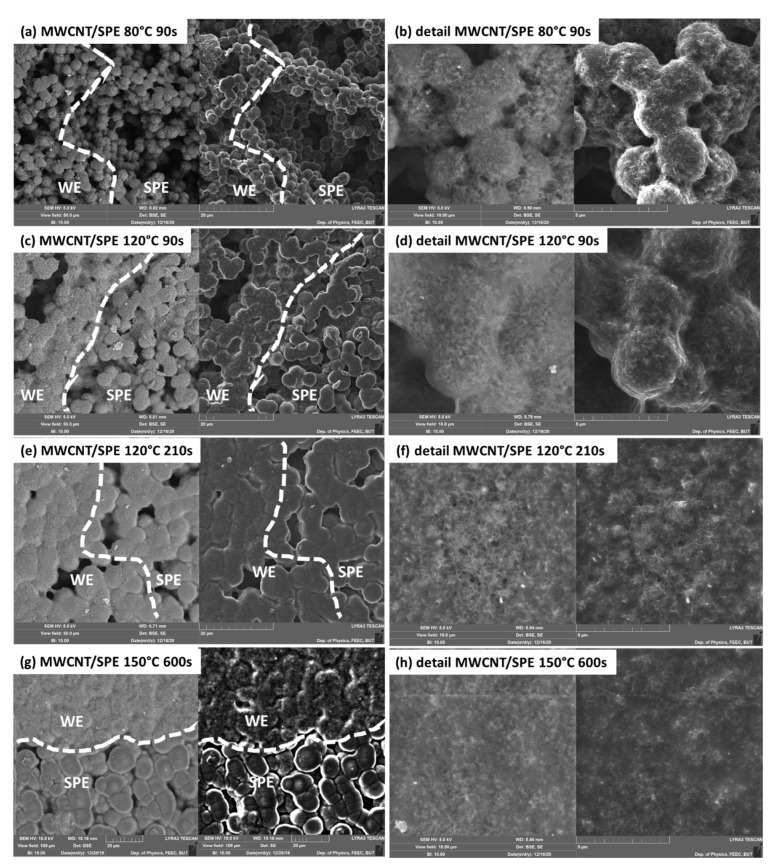
SEM images (back-scattered electron imaging and secondary electron imaging) of the MWCNT WE–SPE interface with the [EMIM][TFSI] ionic liquid prepared for 90 s at 80 °C (**a**,**b**), 90 s at 120 °C (**c**,**d**), 210 s at 120 °C (**e**,**f**) and 600 s at 150 °C (**g**,**h**) with view-field 100 μm (**a**,**c**,**e**,**g**). The boundary lines of WE–SPE are estimated and plotted as dashed lines. (**b**,**d**,**f**,**h**) show details of WE electrodes on SPE layers of corresponding crystallinities with view-field 10 μm.

From the morphological point of view, the interfaces of the MWCNT working electrode and the SPEs based on [EMIM][TFSI] ionic liquid are driven by the varying crystallinity of PVDF in SPE (see Figure 2). As we described in our previous paper [37], the SPE layer consists of so-called spherulites (spherical objects with rougher surfaces, e.g., in Figure 2a). Their size is controlled by thermal treatment conditions (crystallization conditions), i.e., solvent evaporation rate during the preparation of SPE layers (see Table 1, Section 3.3). Their mean size increases from ~3.08 μm up to ~15.12 μm as the solvent evaporation rate grows.

For the SPE treatment with lower temperatures and shorter times (lower solvent evaporation rates), the electrolyte layers are characterized by higher porosity and the presence of smaller spherulites (see Figure 2a–d). This means that the spherulites are almost surrounded by MWCNT, preserving the original SPE microstructure as shown in detail in Figure 2b,d. For the SPE treatment with higher temperatures and longer times (i.e., SPE layers with larger spherulites and lower porosity—higher solvent evaporation rates, Figure 2e–h), MWCNT deposition on the SPE layer creates a carpet-type covering, which is probably caused by the lower porosity of the SPE layers. No clear boundary line of WE–SPE can be observed in the SE and BSE images due to the highly porous microstructure of SPE in Figure 2a. As SPE porosity decreases, the interface becomes clearer, as shown in Figure 2c,e,g.

When PVDF is dissolved in a solvent, very small crystalline entities could still exist in the solution due to partial dissolution of the crystals or refolding of dissolved polymer chains, which serve as nuclei in the recrystallization of the solution [48,49]. These cannot be destroyed with a longer dissolution time but only by raising the dissolution temperature [50]. The formation of spherulites is linked to the phase separation process between the solvent and the polymer during crystallization, and their spherulitic structure strongly depends on whether a particular spherulite emerges through homogeneous or heterogeneous nucleation [51,52]. Generally, the preparation of the SPE layers at higher temperatures and longer times results in the SPE undergoing a reduction of surface tension, and it spreads more evenly, gradually reducing the number of spherulites, which creates a layer with very low porosity. It can be reasonably said that the effective area of the WE–SPE interface changes with the different microstructure (crystallinity) of the SPE layer [37] and the particle size of the carbon material of the working electrode (MWCNT). Thus, effective areas of particular interfaces can markedly differ because of the different overall contact surfaces between the working electrode and the SPE [37]. Considering that the effective area of the WE–SPE interface usually represents the total electrochemically active area where the chemical reaction occurs (reduction of the NO_2_ molecules for our sensor), such an interface should influence the sensitivity of the sensor.

**Figure 3:**
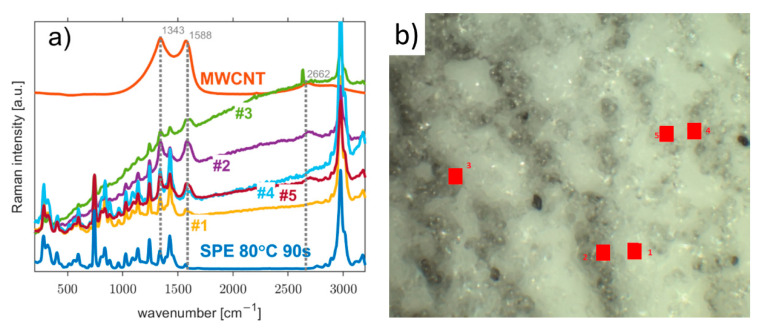
(**a**) Raman spectra of the MWCNT working electrode and solid polymer electrolyte (SPE treated at 80 °C for 90 s) and their mutual interface corresponding to marked positions from #1 to #5 at (**b**) confocal microscopy image of their acquisition locations.

In the Raman spectrum of the carbon working electrode (the white square Figure 1a), the two most important peaks are at around 1343 cm^−1^ and 1588 cm^−1^, as seen in Figure 3a (orange curve). These peaks can be assigned to the D-band and G-band of the carbon material, respectively [53]. The second most prominent peaks are located around 2662 cm^−1^, representing the G’(2D) -band, and 2912 cm^−1^, representing the D+G -band. Figure 3a also shows Raman spectra for several measurement locations at the WE–SPE interface and also the Raman spectrum of the SPE treated at 80 °C for 90 s, which was described in detail in our previous paper [37]. While there seems to be no immediately obvious new information, a more detailed inspection reveals a new factor in the form of a slowly rising background signal.

This rising signal can be attributed to fluorescence, which could indicate an increasing number of vacancies in the measured locations of the interface of the SPE and the working electrode, which indicates the transition between the two materials. The main peaks for the D-band and G-band at 1343 cm^−1^ and 1588 cm^−1^, respectively, are still present and quite visible. The 2662 cm^−1^ peak for the G’-band is slowly drowned out in the fluorescence background, and the areas around 741 cm^−1^ and 2989 cm^−1^ are dominated by peaks belonging to SPE, especially at measurement locations with a thinning working electrode layer, as mentioned previously. All of the spectra measured at the interface were normalized at 741 cm^−1^, corresponding with the ionic liquid in the SPE [37]. Figure 3b shows an SEM image of the scanned areas to illustrate the surface situation on the sample.

### 3.2. Description of the Sensor by Equivalent Electric Circuit

**Figure 4:**
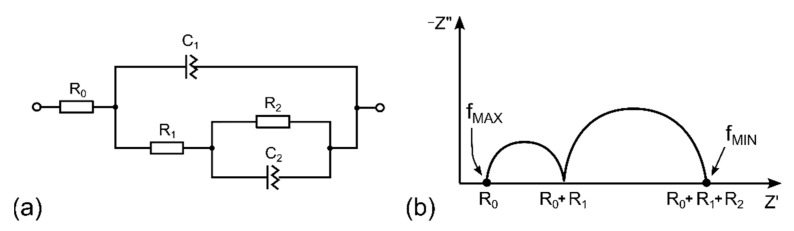
(**a**) Equivalent electric circuit of the NO_2_ sensor, (**b**) typical impedance complex plane (Nyquist) plot for a system with two well-separated time constants.

Electrochemical impedance spectroscopy (EIS) is a universal and nondestructive technique that allows observation of the response of the studied system (sample) over a wide frequency range and description of the sample by an equivalent electric circuit (EEC). Figure 4 shows the equivalent electric circuit designed for the presented NO_2_ sensor (4a) and an ideal complex impedance plot for the circuit with two well-separated time constants (4b).

The EEC in Figure 4a is based on the Randles circuit [54], although the presented electrochemical sensor (cell) does not contain a standard liquid electrolyte with solvated ions where a large excess of supporting (indifferent) electrolyte can be present. For the sake of simplicity of the EEC, the particular discrete components in Figure 4a are as follows: R_0_ is the series resistance of the carbon-based working electrode, R_1_ is the bulk resistance of the solid polymer electrolyte (SPE) layer, C_1_ is the bulk (geometrical) capacitance of the WE–SPE–CE structure (see the cross-section in Figure 1a), R_2_ is the interfacial resistance (which can generally combine various contributions from several processes such as charge transfer resistance, adsorption and mass-transfer), C_2_ is the capacitance at the WE–SPE interface. It should be noted that although C_1_ a C_2_ are called capacitance, they are usually replaced by constant phase elements (CPE) in the EEC because of non-ideal properties/processes in the studied system, such as inhomogeneity in the SPE bulk and inhomogeneity, roughness and porosity of the WE [55].

In contrast to the vast majority of electrochemical cells, the working electrode resistance (R_0_) cannot be neglected in the presented sensor because the carbon WE is very thin, and the resistance of such an electrode can strongly depend on the deposition process. The bulk properties of any electrochemical cell (R_1_ and C_1_) are usually outside the standard measured frequency range because of the very low time constant (τ_1_ = R_1_·C_1_), which requires the application of test frequencies in the range of MHz. However, the solid polymer electrolyte (SPE) showed higher resistance than its liquid counterparts, and the parallel electrode arrangement WE–SPE–CE (see the cross-section in Figure 1a) with the SPE layer thickness of approximately 200 µm allowed the reasonable assumption that the time constant τ_1_ is within the usual tested range (0.1 MHz–0.01 mHz).

**Figure 5:**
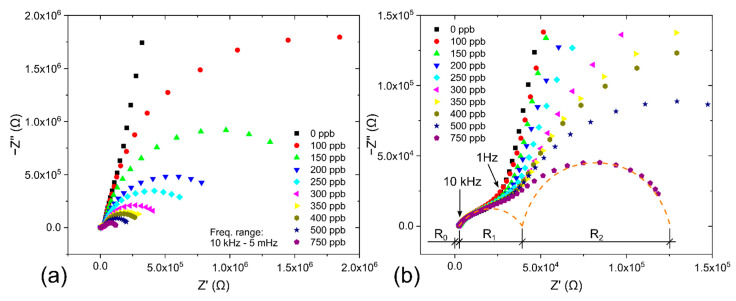
Nyquist plots for the NO_2_ sensor (SPE treated at 80 °C for 90 s) with MWCNT-based working electrode for different NO_2_ concentrations (operation conditions: 298 K, 40%RH, 1013.25 hPa, analyte flow rate 1 L/min, V_BIAS_= −0.5 V): (**a**) Nyquist plot for the whole tested frequency range: 10 kHz–5 mHz, (**b**) Detail of the Nyquist plot for higher frequencies.

**Figure 6:**
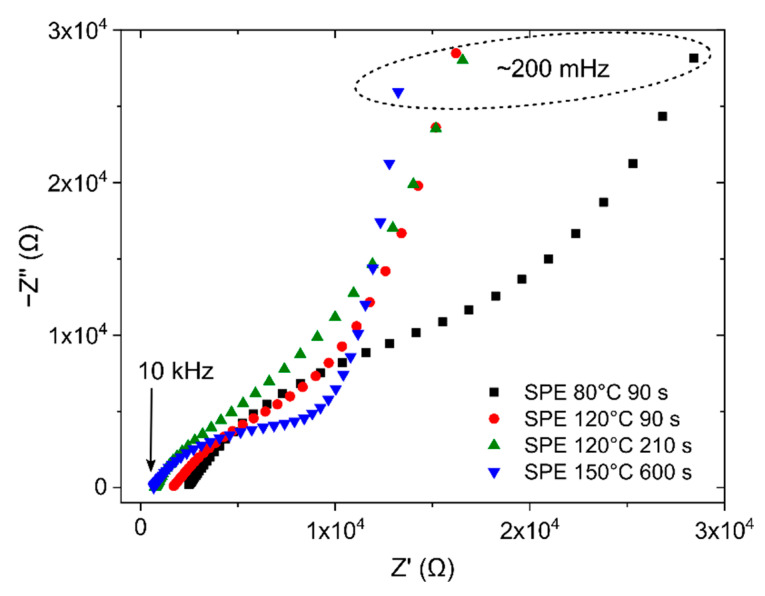
Nyquist plot for NO_2_ sensors with different SPE types for 0 ppm NO_2_.

This assumption was experimentally confirmed by the EIS measurement when two not well-separated arcs were observed in the Nyquist plot (see Figure 5a, where the first small arc is not visible because of the inappropriate scale of both axes). The position and shape of the first depressed arc changed as the geometry of the WE–SPE–CE structure changed (e.g., with the different thickness and/or area of the WE and different microstructural properties and thickness of the SPE layer, see Figure 6) while remaining almost the same with different NO_2_ concentrations (see Figure 5b). While the first small arc was influenced by the cell geometry, the shape and size of the second arc (at frequencies below 1 Hz) changed with the presence of gaseous NO_2_. These experimental results suggest that the R_2_ and C_2_ elements are associated with processes at the electrode–electrolyte interface. For simplicity, let us consider that C_2_ represents the interfacial capacitance WE–SPE, and interfacial resistance R_2_ predominantly represents only the charge transfer resistance of the electrochemical reaction on the WE–SPE interface (i.e., reduction of the NO_2_ molecule). In the absence of NO_2_, when there is no reaction on the WE–SPE interface (i.e., no electron transfer), the charge transfer resistance becomes very large (ideally infinitely large), and capacitive behavior dominates at low frequencies. In the presence of NO_2_, charge transfer resistance becomes smaller, resulting in parallel R_2_ and C_2_ connections in the EEC. However, it should be pointed out that real mechanisms at the electrode–electrolyte interface can be more complicated than in the simplified EEC in Figure 4a. The interfacial resistance R_2_ can be further divided into more discrete elements representing other low-frequency processes, such as diffusion and/or adsorption of electroactive species in close proximity to the electrode–electrolyte interface [56].

Concerning the effect of solvent evaporation rate, Figure 6 shows EIS spectra within the frequency range from 10 kHz to 200 mHz for sensors with different types of SPE at zero NO_2_ concentration. 

Experimental results show that the SPE type has an impact on the position and shape of the first depressed arc, which should be represented by the SPE resistance and geometrical capacitance of the sensor itself. The shift of the spectra to the left, i.e., the decrease of the WE resistance (R_0_ in EEC), was observed for SPE layers treated at higher temperatures and longer times (SPE layers with lower porosity). The results indicate that higher SPE porosity causes higher WE resistance because the working electrodes for all the samples in Figure 6 were prepared with the same number of deposited MWCNT layers. Intersection points of the spectra with the real axis were found to be 0.66, 0.74, 1.68 and 2.41 kΩ for SPE conditions 150 °C for 600 s, 120 °C for 210 s, 120 °C for 90 s and 80 °C for 90 s respectively. A possible explanation is that SPE layers with low porosity allow the deposition of a more compact and homogeneous WE layer with lower resistance. The size and shape of the depressed arc are associated with the SPE resistance and geometrical capacitance. The biggest arc for the SPE layer at 80 °C for 90 s represents the highest SPE resistance which correlates with the lowest SPE conductivity for such conditions in Table 1. The smallest arc for the SPE layer at 150 °C for 600 s represents the lowest SPE resistance which correlates with the highest SPE conductivity for such conditions in Table 1.

### 3.3. Effect of Solvent Evaporation Rate during SPE Preparation on the Performance of an Amperometric Gas Sensor

The solvent evaporation (thermal treatment conditions) during the preparation of the electrolyte layer drives the PVDF crystallinity and thus changes the porosity of the microstructure (i.e., diameter of spherulites) of the [EMIM][TFSI] based SPE layer [37] as well as its electrical conductivity [9]. The porosity of the SPE layer decreases with the increasing diameter of the spherulites [37] (as the solvent evaporation rate increases), while the direct current conductivity of the SPE layer becomes comparable to the direct current conductivity of its component, i.e., the ionic liquid [EMIM][TFSI]. This is because the ion transport takes place predominantly on the surfaces of the spherulites as well as in the amorphous regions [37]. These facts raise the question of how SPE spherulite diameter (i.e., SPE microstructure) and SPE conductivity affect the performance of the MWCNT/SPE-based amperometric sensor.

Table 1 summarizes the direct current conductivity and spherulite diameter of four variants of solid polymer electrolytes, which were published in our previous studies [9,37]. It should be noted that all our previous studies on SPEs were performed on SPE samples prepared with treatment conditions of 80 °C for 90 s, 120 °C for 90 s, 120 °C for 210 s and 160 °C for 600 s. However, sensors with the SPE layers treated at the highest temperature in this study were prepared at 150 °C instead of 160 °C for purely technological reasons. We have taken the liberty of replacing the conductivity and spherulite size values of the SPE layers prepared at 150 °C with those prepared at 160 °C to demonstrate the nature of the effect of solvent evaporation on sensor performance.

#### 3.3.1. Effect on Sensitivity, Signal-to-Noise Ratio, Limit-of-Detection and Saturation Current

**Figure 7:**
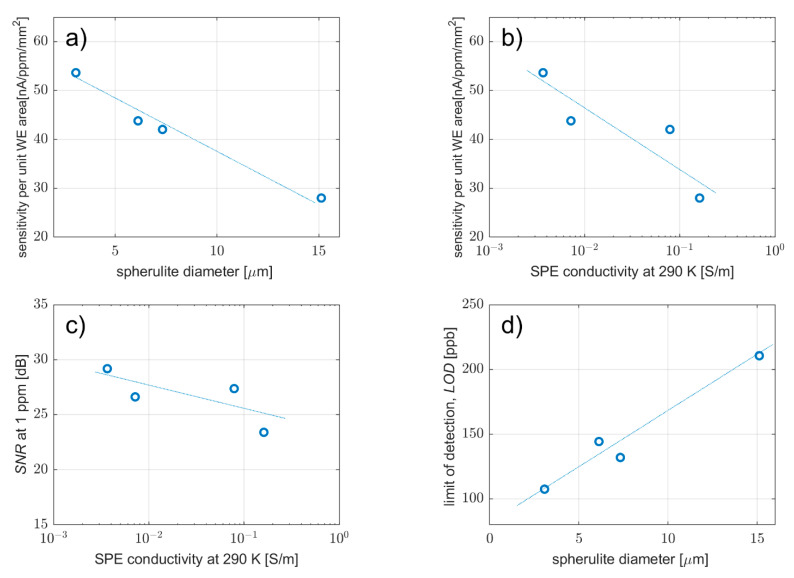
Effect on sensor properties of solvent evaporation rate from solid polymer electrolyte during preparation: (**a**) sensor sensitivity per unit WE area as a function of electrolyte-spherulite diameter, (**b**) sensor sensitivity per unit WE area as a function of electrolyte conductivity, (**c**) signal-to-noise ratio as a function of electrolyte conductivity, (**d**) limit-of-detection as a function of electrolyte-spherulite diameter.

Sensitivity is one of the fundamental parameters of any gas sensor. Sensitivity expresses the change of an output quantity on the input conditions due to a change in the physical structure or chemical properties of an active layer or active interface. The sensitivities of all the sensors were determined as the slope of their calibration curves in the linear region (see Supplementary Materials and Figure S2). To remove possible inaccuracies resulting from the effect of the geometric area of the WE electrode on the sensor sensitivity, the sensitivity of each type was converted to unit area. As can be seen in Figure 7a,b, sensor sensitivity per unit WE area with the MWCNT working electrode shows a steep decrease as the spherulite diameter increases (decreasing SPE porosity), as well as with SPE conductivity. For the SPE layer with high porosity, small SPE spherulites can be surrounded by MWCNTs, and the mutual contact surface (active area) can be maximized. As the diameter of the spherulites increases, the porosity of the SPE layer decreases, which probably goes hand in hand with the decrease of the electrochemically active area of the MWCNT–SPE interface, which results in the decrease of the sensor sensitivity.

The signal-to-noise ratio (SNR) compares the level of the desired output signal with the level of the undesired signal. Here the parameter is calculated as a logarithmic ratio between the average value of steady-state current at 1 ppm NO_2_ and the standard deviation of its fluctuation at 1 ppm NO_2_. As far as a signal-to-noise ratio (SNR) is concerned, the measurements showed very small changes with increasing conductivity (spherulite diameter) (Figure 7c). Higher current flowing through the sensor at 1 ppm NO_2_ should increase the SNR parameter, but it also means that more NO_2_ molecules take part in the adsorption-desorption process on the WE–SPE interface, which in turn increases the noise in the system. The antagonistic character of these processes is responsible for the negligible dependence of the SNR parameter on the different spherulite diameters (Table 1).

The limit-of-detection (LOD) represents the lowest concentration of gas that can be identified consistently and is calculated as the ratio of the triple standard deviation of the current background noise (at zero concentration) and sensitivity. Because the LOD is inversely proportional to sensor sensitivity, LOD vs. spherulite diameter dependence (Figure 7d) exhibits the opposite trend to that shown in Figure 7a. Thus, the lowest LOD was calculated for the MWCNT working electrode with the SPE layer prepared at 80 °C for 90 s. It should be noted that the LOD parameter is very sensitive to the way the standard deviation of background current is measured and estimated. Data filtering and the low sampling rate of data acquisition can cause this value to drop by orders of magnitude. The estimated LOD values in Figure 7d are calculated from the current fluctuation spectral density data and are generally higher than for signals where these current fluctuations are considered undesirable and thus suppressed.

While the LOD determines theoretically the lowest detectable level of the analyte, the highest level is usually restricted by the saturation of the current response (*I_SAT_*) that is no longer increased in spite of the growth of analyte concentration. This situation can be explained by the lack of free adsorption sites on the WE–SPE interface, i.e., full coverage of the surface of the interface by analyte molecules. Thus, saturation current can be predicted by fitting the calibration curve by the Langmuir adsorption isotherm (1), assuming the steady-state current response of the sensor is proportional to the fractional surface coverage of the WE–SPE interface [40].

(1) $$I=I_{SAT}\;Ap/\left(1+Ap\right)$$

where *I* is the steady-state current response, *I_SAT_* is the saturation current, *p* is the partial pressure, and *A* is a constant.

**Figure 8:**
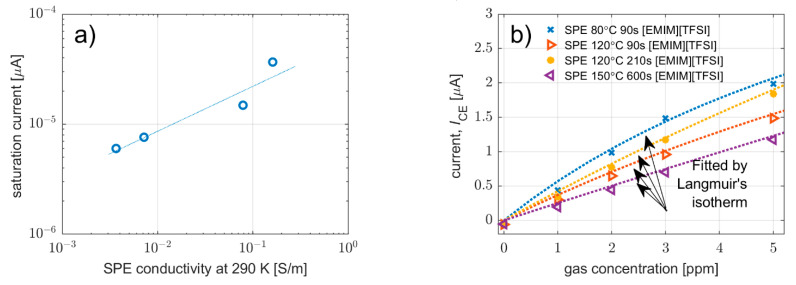
(**a**) Saturation current of sensor response as a function of SPE conductivity. (**b**) Current response as a function of NO_2_ concentration for three sensors prepared with various crystallinities (i.e., of various conductivities). The measured points were fitted by a function derived from the Langmuir adsorption isotherm.

Figure 8a shows the dependence of saturation current on SPE conductivity obtained from fitting the analysis of particular calibration curves presented in Figure 8b. These results indicate that the level of saturation current is influenced by SPE conductivity; the lower the SPE conductivity, the lower the saturation current. It should be noted that the authors were able to perform the fitting analysis based on the Langmuir adsorption isotherm without any doubt due to previous studies [40,45], where similar sensors were tested over a wider concentration range.

#### 3.3.2. Effect on Current Fluctuations

**Figure 9:**
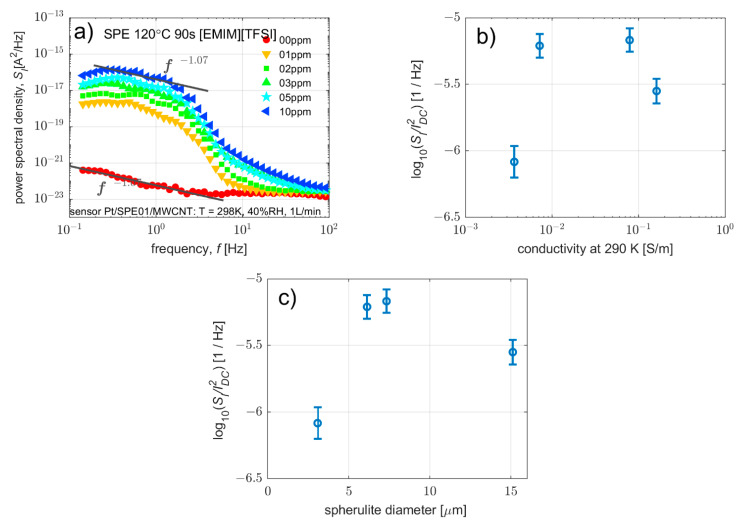
Effect of carbon working electrode and polymer electrolyte with various microstructures on current fluctuations of particular sensor: (**a**) Spectral density of current fluctuations for all measured NO_2_ concentrations (sensor with SPE prepared at 120 °C for 90 s), (**b**) ratio of the spectral density of current fluctuations at 1 Hz to the square of the direct current as a function of electrolyte conductivity, (**c**) ratio of spectral density of current fluctuations at 1 Hz to the square of the direct current as a function of electrolyte—spherulite diameter.

In this section, the influence of SPE conductivity on the spectral density of current fluctuations is discussed. Figure 9a shows the typical shapes of the characteristics of the spectral densities of the current fluctuations that were observed for all the samples (i.e., for all types of SPE). At zero concentration of the detected substance, the spectral density of the current fluctuations is dominated by 1/*f* noise, which can indicate a contact noise, or a diffusion–dominant interface between SPE and WE [9,41,57], or also thermal noise associated with the dissipative processes in the sensor. In the case of non-zero concentrations of the detected substance, a characteristic spectrum can be observed [40,41,42,44], containing thermal noise, an *f*^−2^ noise component indicating adsorption-desorption or a drift-dominant electrode–electrolyte interface [57] or a noise component of Lorentzian-like spectra assumed to be the result of several mutually influencing stochastic processes [41], combining 1/*f*, diffusion and adsorption-desorption noises with disturbances caused by partial pressure fluctuations [58] and velocity fluctuations [59] as a result of analyte flow around the sensor.

The samples yield different levels of current fluctuations. In order to compare the level of fluctuations of the adsorbed molecules on the WE–SPE interface, the ratio of the spectral density of current fluctuations at 1 Hz to the square of the direct current is determined. The frequency of 1 Hz was selected with regards to the change of spectral density of current fluctuations for detected substance concentrations from 1 ppm to 10 ppm. Figure 9b shows that the *S*_I_/*I*^2^ ratio remains nearly constant within one order of magnitude compared to a change in conductivity of more than two orders of magnitude. The increase in spherulite diameter is shown in Figure 9c. This indicates, as mentioned above that the main source of noise is associated with charge transport at the electrode–electrolyte interface [57].

#### 3.3.3. Complete Summary of Sensor Parameters

In contrast to Section 3.3.1, where very sensitive laboratory apparatus operating at high sampling rates was used for sensor characterization, this section aims at a general overview of the effect of the SPE type on sensor parameters based on more tested samples. A group of six sensors was prepared for each SPE type, and these sensors were measured simultaneously in the test chamber. The sensors underwent two specific test cycles to determine basic sensor parameters such as sensitivity per unit WE area, LOD, LOQ, repeatability, hysteresis and response/recovery time (see Table 2 where the average value for each parameter is presented). A detailed description of the test cycles and determination of the sensor parameters can be found in the Supplementary Materials (see overall evaluation in Figure S3). It can be generally said that the SPE type primarily influenced the sensitivity and associated parameters such as LOD and LOQ. The other parameters do not show any significant trend with respect to the SPE type. It is important to note that the results of the LOD parameter, which was determined in the same way in the two sections (Section 3.3.1 and Section 3.3.3), differ markedly from each other. Both measurements were carried out with different types of potentiostat circuits, and different sampling frequencies (f_SAMPL1_ = 10 kHz in Section 3.3.1 and f_SAMPL2_ = 0.5 Hz in Section 3.3.3), which resulted in different levels of background current noise and so different LOD values were obtained from each measurement, although sensor sensitivities in both measurements were comparable.

## 4. Conclusions

The paper discusses the role of solvent evaporation rate on the overall performance of an amperometric gas sensor. The polymer electrolytes were prepared as a mixture of the polymer host PVDF, solvent NMP and ionic liquid [EMIM][TFSI], and this mixture was thermally treated to achieve different solvent evaporation rates that resulted in various structural and electrical properties of the polymer electrolyte, namely morphology and conductivity as we described in our previous studies[9,37].

Morphology analysis of the working electrode–solid polymer electrolyte interface showed that deposition of a carbon nanotube-based working electrode guaranteed that the prepared electrode–electrolyte interface is driven by the microstructure of polymer electrolyte, which consists of semi-crystalline spherulites. Solvent evaporation rate affected the size of these spherulites and the porosity of the electrolyte, which ultimately affects how much the surface layer of spherulites is coated with multi-wall carbon nanotubes. Lower evaporation rates bring higher porosity of the polymer electrolyte.

Analyses of sensors response revealed that the change of solvent evaporation rate during the preparation of the SPE layer from 80 °C for 90 s to 150 °C for 600 s resulted in an almost 50% decrease in sensor sensitivity. It needs to be pointed out that this reduction of the sensitivity by half is accompanied by an increase of electrical conductivity in the electrolyte by almost two orders of magnitude. Thus, considering the change of electrical conductivity in particular SPE layers, we are convinced that this decrease in sensitivity is related to the size of the effective areas of the WE–SPE interfaces, as the porosity of each interface decreases with an increasing evaporation rate. Also, the electrochemical impedance spectroscopy showed a decrease in WE resistance with decreasing SPE porosity. Since the limits of detection and quantification are inversely proportional to sensitivity, they are also strongly dependent on the effective area of the WE–SPE interface.

The maximum level of current response was predicted by fitting the calibration curves with a Langmuir adsorption isotherm. The higher solvent evaporation rate leads to an increase in the maximum current flowing through the sensor. The level of this saturation current is assumed to be driven by SPE conductivity, which seems to be the limiting factor for the current response under high concentrations of the analyte.

The other properties of the sensor change insignificantly with respect to solvent evaporation rate with the average response/recovery time of 55/22 s, repeatability of 1.5%, and maximum hysteresis below 5%. These values are comparable with commercial amperometric gas sensors.

The noise properties of the tested sensors were not significantly influenced by the different properties of the SPE layers (microstructure and conductivity). As the ratio of power spectral density of current fluctuations to direct current shows, the main source of noise is associated with charge transport at the electrode–electrolyte interface.

Further characterization using electrochemical impedance spectroscopy allowed us to obtain a complete picture of the WE–SPE interface in the sensor and its influence on the sensor parameters. The typical electrical impedance of the sensor in the Nyquist plot exhibits a system of two time constants, represented by two arcs, where the long time constant is associated with processes at the electrode–electrolyte interface, while a short time constant is associated with the properties of sensor itself.

## Figures and Tables

**Table 1:** Direct current conductivity and spherulite diameter of solid polymer electrolyte based on [EMIM][TFSI] ionic liquid, PVDF as polymer matrix and NMP as a solvent and prepared under different conditions.

Preparation Condition	DC Conductivity [S/m]	Mean Spherulite Diameter [μm]
80 °C 90 s	0.003647	3.08
120 °C 90 s	0.007162	6.13
120 °C 210 s	0.078600	7.33
160 °C 600 s	0.161200	15.12

**Table 2:** Sensor parameters.

Parameter	SPE at 80 °C for 90 s	SPE at 120 °C for 90 s	SPE at 120 °C for 210 s	SPE at 150 °C for 600 s
Sensitivity (pA/ppb/mm^2^)	55.0	42.0	31.9	30.6
LOD (ppb)	1.07	1.36	1.72	2.04
LOQ (ppb)	3.57	4.53	5.73	6.78
Hysteresis (%) *	3.9	4.2	4.9	3.9
Repeatability (%)	1.2	2.4	1.3	1.3
Response time (s)	47	58	59	55
Recovery time (s)	22	22	23	21

* Maximum value from six sensor samples within the tested range.

## Data Availability

The datasets measured and analyzed during the current study are available from the corresponding author upon reasonable request.

## Supplementary Materials

The following supporting information can be downloaded at: https://www.mdpi.com/article/10.3390/polym14214758/s1, Figure S1: Sensor response to 10 consecutive exposures to 200 ppb; Figure S2: Sensor response to a stepwise increase and subsequent decrease in NO_2_ concentration; Figure S3: The effect of the SPE type on sensor parameters.

## References

[1] Ye Y.-S., Rick J., Hwang B.-J. (2013). Ionic liquid polymer electrolytes. J. Mater. Chem. A.

[2] Correia D.M., Fernandes L.C., Martins P.M., García-Astrain C., Costa C.M., Reguera J., Lanceros-Méndez S. (2020). Ionic Liquid–Polymer Composites: A New Platform for Multifunctional Applications. Adv. Funct. Mater..

[3] Josef E., Yan Y., Stan M.C., Wellmann J., Vizintin A., Winter M., Johansson P., Dominko R., Guterman R. (2019). Ionic Liquids and their Polymers in Lithium-Sulfur Batteries. Isr. J. Chem..

[4] Austin Suthanthiraraj S., Johnsi M. (2017). Nanocomposite polymer electrolytes. Ionics (Kiel).

[5] Xia W., Zhang Z. (2018). PVDF-based dielectric polymers and their applications in electronic materials. IET Nanodielectrics.

[6] Kammoun M., Berg S., Ardebili H. (2015). Flexible thin-film battery based on graphene-oxide embedded in solid polymer electrolyte. Nanoscale.

[7] Korotcenkov G., Cho B.K. (2011). Instability of metal oxide-based conductometric gas sensors and approaches to stability improvement (short survey). Sens. Actuators B Chem..

[8] Varshney P.K., Gupta S. (2011). Natural polymer-based electrolytes for electrochemical devices: A review. Ionics (Kiel).

[9] Sedlak P., Gajdos A., Macku R., Majzner J., Sedlakova V., Holcman V., Kuberský P. (2020). The effect of thermal treatment on ac/dc conductivity and current fluctuations of PVDF/NMP/[EMIM][TFSI] solid polymer electrolyte. Sci. Rep..

[10] Wang F., Li L., Yang X., You J., Xu Y., Wang H., Ma Y., Gao G. (2018). Influence of additives in a PVDF-based solid polymer electrolyte on conductivity and Li-ion battery performance. Sustain. Energy Fuels.

[11] Subba Reddy C.V., Chen M., Jin W., Zhu Q.Y., Chen W., Mho S. (2007). Il Characterization of (PVDF + LiFePO_4_) solid polymer electrolyte. J. Appl. Electrochem..

[12] Tjong S.C., Li Y.C., Li R.K.Y. (2010). Frequency and temperature dependences of dielectric dispersion and electrical properties of polyvinylidene fluoride/expanded graphite composites. J. Nanomater..

[13] Puértolas J.A., García-García J.F., Pascual F.J., González-Domínguez J.M., Martínez M.T., Ansón-Casaos A. (2017). Dielectric behavior and electrical conductivity of PVDF filled with functionalized single-walled carbon nanotubes. Compos. Sci. Technol..

[14] Li X., Xuan T., Yin G., Gao Z., Zhao K., Yan P., He D. (2015). Highly sensitive amperometric CO sensor using nanocomposite C-loaded PdCl_2_–CuCl_2_ as sensing electrode materials. J. Alloys Compd..

[15] Tofel P., Částková K., Říha D., Sobola D., Papež N., Kaštyl J., Ţălu Ş., Hadaš Z. (2022). Triboelectric Response of Electrospun Stratified PVDF and PA Structures. Nanomaterials.

[16] Černohorský P., Pisarenko T., Papež N., Sobola D., Ţălu Ş., Částková K., Kaštyl J., Macků R., Škarvada P., Sedlák P. (2021). Structure Tuning and Electrical Properties of Mixed PVDF and Nylon Nanofibers. Materials.

[17] Nespurek S., Mracek L., Kubersky P., Syrovy T., Hamacek A. (2019). Ionic liquids in electrochemical gas sensors and transistors. Mol. Cryst. Liq. Cryst..

[18] Ohno H. (2011). Electrochemical Aspects of Ionic Liquids.

[19] Torriero A.A.J., Shiddiky M.J.A. (2011). Electrochemical Properties and Applications of Ionic Liquids.

[20] Kirchner B., Perlt E. (2018). Ionic Liquids II.

[21] Paluch M. (2016). Dielectric Properties of Ionic Liquids.

[22] Brandt A., Pohlmann S., Varzi A., Balducci A., Passerini S. (2013). Ionic liquids in supercapacitors. MRS Bull..

[23] Kuberský P., Navrátil J., Syrový T., Sedlák P., Nešpůrek S., Hamáček A. (2021). An Electrochemical Amperometric Ethylene Sensor with Solid Polymer Electrolyte Based on Ionic Liquid. Sensors.

[24] Kuberský P., Altšmíd J., Hamáček A., Nešpůrek S., Zmeškal O. (2015). An electrochemical NO_2_ sensor based on ionic liquid: Influence of the morphology of the polymer electrolyte on sensor sensitivity. Sensors.

[25] Luo R., Li H., Du B., Zhou S., Chen Y. (2019). A printed and flexible NO_2_ sensor based on a solid polymer electrolyte. Front. Chem..

[26] Luo R., Li Q., Du B., Zhou S., Chen Y. (2019). Preparation and Characterization of Solid Electrolyte Doped With Carbon Nanotubes and its Preliminary Application in NO_2_ Gas Sensors. Front. Mater..

[27] Luo R., Jiang H., Du B., Zhou S., Zhu Y. (2019). Preparation and application of solid polymer electrolyte based on deep eutectic solvent. AIP Adv..

[28] Nádherná M., Opekar F., Reiter J. (2011). Ionic liquid–polymer electrolyte for amperometric solid-state NO_2_ sensor. Electrochim. Acta.

[29] Strzelczyk A., Jasinski G., Chachulski B. (2016). Investigation of solid polymer electrolyte gas sensor with different electrochemical techniques. IOP Conf. Ser. Mater. Sci. Eng..

[30] Dosi M., Lau I., Zhuang Y., Simakov D.S.A., Fowler M.W., Pope M.A. (2019). Ultrasensitive Electrochemical Methane Sensors Based on Solid Polymer Electrolyte-Infused Laser-Induced Graphene. ACS Appl. Mater. Interfaces.

[31] Satapathy S., Pawar S., Gupta P.K., RVarma K.B. (2011). Effect of annealing on phase transition in poly(vinylidene fluoride) films prepared using polar solvent. Bull. Mater. Sci..

[32] Ting Y., Suprapto, Bunekar N., Sivasankar K., Aldori Y.R. (2020). Using Annealing Treatment on Fabrication Ionic Liquid-Based PVDF Films. Coatings.

[33] Xu P., Fu W., Hu Y., Ding Y. (2018). Effect of annealing treatment on crystalline and dielectric properties of PVDF/PEG-containing ionic liquid composites. Compos. Sci. Technol..

[34] Gregorio R., Borges D.S. (2008). Effect of crystallization rate on the formation of the polymorphs of solution cast poly(vinylidene fluoride). Polymer.

[35] Dong Z., Zhang Q., Yu C., Peng J., Ma J., Ju X., Zhai M. (2013). Effect of ionic liquid on the properties of poly(vinylidene fluoride)-based gel polymer electrolytes. Ionics (Kiel).

[36] Correia D.M., Costa C.M., Rodríguez-Hernández J.C., Tort Ausina I., Biosca L.T., Torregrosa Cabanilles C., Meseguer-Duenãs J.M., Lanceros-Méndez S., Gomez Ribelles J.L. (2020). Effect of Ionic Liquid Content on the Crystallization Kinetics and Morphology of Semicrystalline Poly(vinylidene Fluoride)/Ionic Liquid Blends. Cryst. Growth Des..

[37] Sedlak P., Sobola D., Gajdos A., Dallaev R., Nebojsa A., Kubersky P. (2021). Surface analyses of PVDF/NMP/[EMIM][TFSI] solid polymer electrolyte. Polymers.

[38] Altšmíd J., Syrový T., Syrová L., Kuberský P., Hamáček A., Zmeškal O., Nešpůrek S. (2015). Ionic Liquid based polymer electrolytes for electrochemical sensors. Mater. Sci..

[39] Luo R., Wu Y., Li Q., Du B., Zhou S., Li H. (2021). Rational synthesis and characterization of IL-CNTs-PANI microporous polymer electrolyte film. Synth. Met..

[40] Kuberský P., Sedlák P., Hamáček A., Nešpůrek S., Kuparowitz T., Šikula J., Majzner J., Sedlaková V., Grmela L., Syrový T. (2015). Quantitative fluctuation-enhanced sensing in amperometric NO_2_ sensors. Chem. Phys..

[41] Sedlák P., Kuberský P., Mívalt F. (2019). Effect of various flow rate on current fluctuations of amperometric gas sensors. Sens. Actuators B Chem..

[42] Sedlák P., Kuberský P. (2020). The Effect of the Orientation Towards Analyte Flow on Electrochemical Sensor Performance and Current Fluctuations. Sensors.

[43] Kuberský P., Hamáček A., Nešpůrek S., Soukup R., Vik R. (2013). Effect of the geometry of a working electrode on the behavior of a planar amperometric NO_2_ sensor based on solid polymer electrolyte. Sens. Actuators B Chem..

[44] Sedlak P., Kubersky P., Skarvada P., Hamacek A., Sedlakova V., Majzner J., Nespurek S., Sikula J. (2016). Current Fluctuation Measurements of Amperometric Gas Sensors Constructed with Three Different Technology Procedures. Metrol. Meas. Syst..

[45] Kuberský P., Syrový T., Hamáček A., Nešpůrek S., Syrová L. (2015). Towards a fully printed electrochemical NO_2_ sensor on a flexible substrate using ionic liquid based polymer electrolyte. Sens. Actuators B Chem..

[46] Ahmadi M.M., Jullien G.A. (2009). Current-Mirror-Based Potentiostats for Three-Electrode Amperometric Electrochemical Sensors. IEEE Trans. Circuits Syst. I Regul. Pap..

[47] Cui Z., Hassankiadeh N.T., Zhuang Y., Drioli E., Lee Y.M. (2015). Crystalline polymorphism in poly(vinylidenefluoride) membranes. Prog. Polym. Sci..

[48] California A., Cardoso V.F., Costa C.M., Sencadas V., Botelho G., Gómez-Ribelles J.L., Lanceros-Mendez S. (2011). Tailoring porous structure of ferroelectric poly(vinylidene fluoride-trifluoroethylene) by controlling solvent/polymer ratio and solvent evaporation rate. Eur. Polym. J..

[49] Magalhães R., Durães N., Silva M., Silva J., Sencadas V., Botelho G., Gómez Ribelles J.L., Lanceros-Méndez S. (2010). The Role of Solvent Evaporation in the Microstructure of Electroactive β-Poly(Vinylidene Fluoride) Membranes Obtained by Isothermal Crystallization. Soft Mater..

[50] Li C.L., Wang D.M., Deratani A., Quémener D., Bouyer D., Lai J.Y. (2010). Insight into the preparation of poly(vinylidene fluoride) membranes by vapor-induced phase separation. J. Memb. Sci..

[51] Crist B., Schultz J.M. (2016). Polymer spherulites: A critical review. Prog. Polym. Sci..

[52] Sheiko S.S., Magonov S.N., Martin Moeller K.M. (2012). Scanning Probe Microscopy of Polymers. Polymer Science: A Comprehensive Reference.

[53] Noor N.A.M., Kamarudin S.K., Darus M., Yunos N.F.D.M., Idris M.A. (2018). Photocatalytic Properties and Graphene Oxide Additional Effects in TiO_2_. Solid State Phenom..

[54] Randles J.E.B. (1947). Kinetics of rapid electrode reactions. Faraday Discuss..

[55] Lvovich V.F. (2015). Impedance spectroscopy: Applications to electrochemical and dielectric phenomena.

[56] Chang S.-C., Stetter J.R. (1990). Electrochemical NO_2_ gas sensors: Model and mechanism for the electroreduction of NO_2_. Electroanalysis.

[57] Hassibi A., Navid R., Dutton R.W., Lee T.H. (2004). Comprehensive study of noise processes in electrode electrolyte interfaces. J. Appl. Phys..

[58] Musha T., Higuchi H. (1978). Traffic Current Fluctuation and the Burgers Equation. Jpn. J. Appl. Phys..

[59] Roach P.E. (1987). The generation of nearly isotropic turbulence by means of grids. Int. J. Heat Fluid Flow.

