# Supplementary material for: Solvent Evaporation Rate as a Tool for Tuning the Performance of a Solid Polymer Electrolyte Gas Sensor
Source: Polymers (Basel). 2022 Nov 6;14(21):4758. doi: 10.3390/polym14214758 (PMC9658810; doi:10.3390/polym14214758)
Supplement: Supplementary file 1 [file polymers-14-04758-s001.zip › polymers-1985625-supplementary.pdf]

Solvent evaporation rate as tool for tuning the performance of solid-polymer-electrolyte gas sensor

Petr Sedlak, Pavel Kaspar, Dinara Sobola, Adam Gajdos, Jiri Majzner, Vlasta Sedlakova, and Petr Kubersky

In order to obtain a general overview of the effect of the SPE type (i.e. SPE prepared under different thermal treatment conditions) on sensor parameters, six sensors were prepared for each SPE type. Each group of six samples were exposed to two basic test cycles. The first one consisted of ten consecutive exposures to the same level of NO_2 concentration (example of the sensor response is shown in Fig. S1). Such test cycle allowed to determine response/recovery time and repeatability of the sensor response. Response time was defined as a time period which was necessary to achieve 90 % of the full current response upon a step increase in the NO_2 concentration. Analogically, recovery time was defined as a time period which was necessary to achieve 10 % of the full current response upon a step decrease in the NO_2 concentration. Repeatability of the sensor response was defined as a relative standard deviation from 10 consecutive exposures when the level of the response for each exposure (red circles in Fig. S1) was calculated as an average value from the last minute of each exposure (i.e. from last 30 data points) where the sensor response achieved a steady state. The second test cycle was represented by a stepwise increase and subsequent decrease in NO_2 concentration within the range of 0–1 ppm with the step height of 0.1 ppm (example of the sensor response is shown in Fig. S2). Such test cycle allowed to construct a calibration curve and determine sensitivity, limit of detection (LOD), limit of quantification (LOQ) and hysteresis of the sensor response. Sensitivity was determined as the slope of the calibration curve when each level of current response for particular concentration (red circles in Fig. S2) was calculated as an average value from the last minute of the exposure to the particular concentration. The limit of detection (LOD) was calculated as the ratio of the triple standard deviation of the background current noise (at zero concentration) and sensitivity. The limit of quantification (LOQ) was calculated as the ratio of the ten times standard deviation of the background current noise (at zero concentration) and sensitivity.

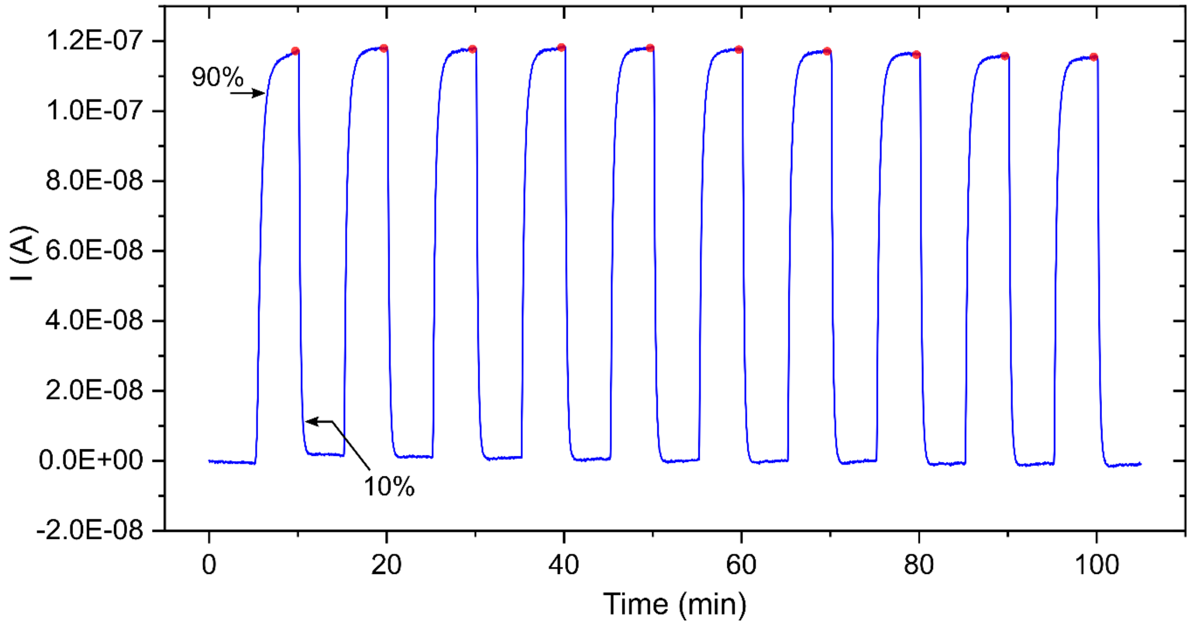

Figure S1. Sensor response to 10 consecutive exposures to 200 ppb NO_2 (conditions: 298 K, 40%RH, 1013.25 hPa, 1 L/min analyte flow rate, sensor with SPE prepared at 80 °C for 90 s).

The hysteresis of the sensor was calculated as the maximum difference in the output currents obtained at the same level of NO_2 exposure, the first current value obtained when the NO_2 concentration was

increasing, the second obtained when the NO_2 concentration was decreasing. The measured difference was related to the upper limit of the measured range and expressed in percentage.

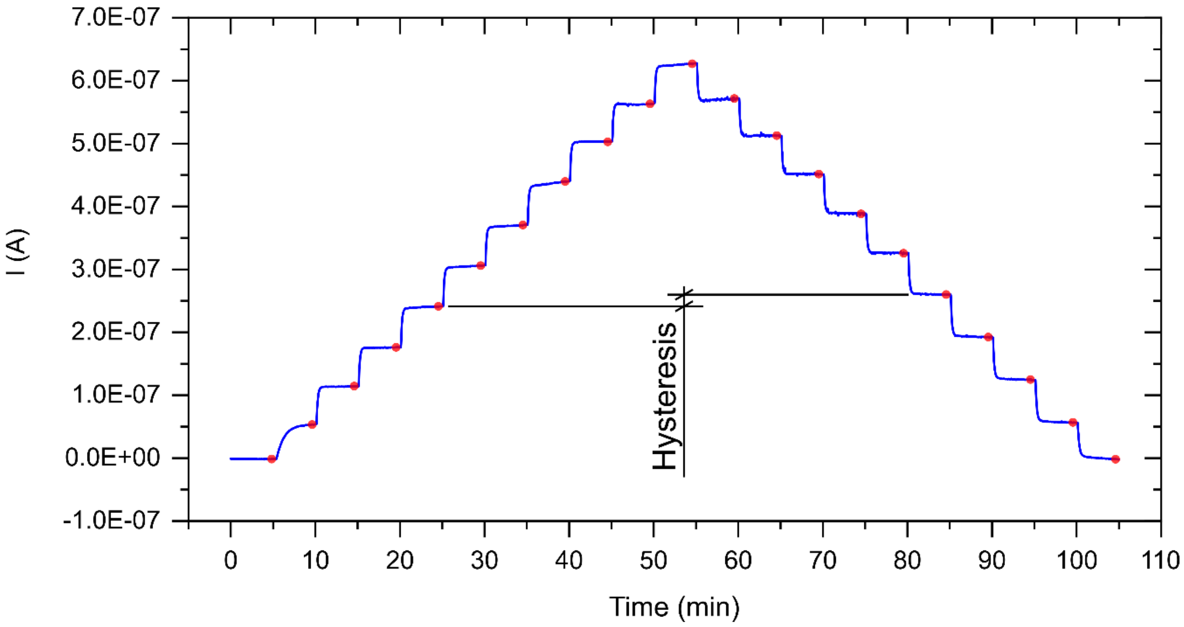

Figure S2. Sensor response to a stepwise increase and subsequent decrease in NO_2 concentration within the range of 0–1 ppm with the step height 0.1 ppm (conditions: 298 K, 40%RH, 1013.25 hPa, 1 L/min analyte flow rate, sensor with SPE prepared at 80 °C for 90 s).

The summary of the effect of the SPE type, i.e. SPE prepared under different thermal treatment conditions, on sensor performance is shown in Fig. S3. In order to compare detection capability of sensors with different SPE types that have slight differences WE areas, the sensitivity was divided by the WE geometric area of the particular sample in order to obtain sensitivity per unit WE area.

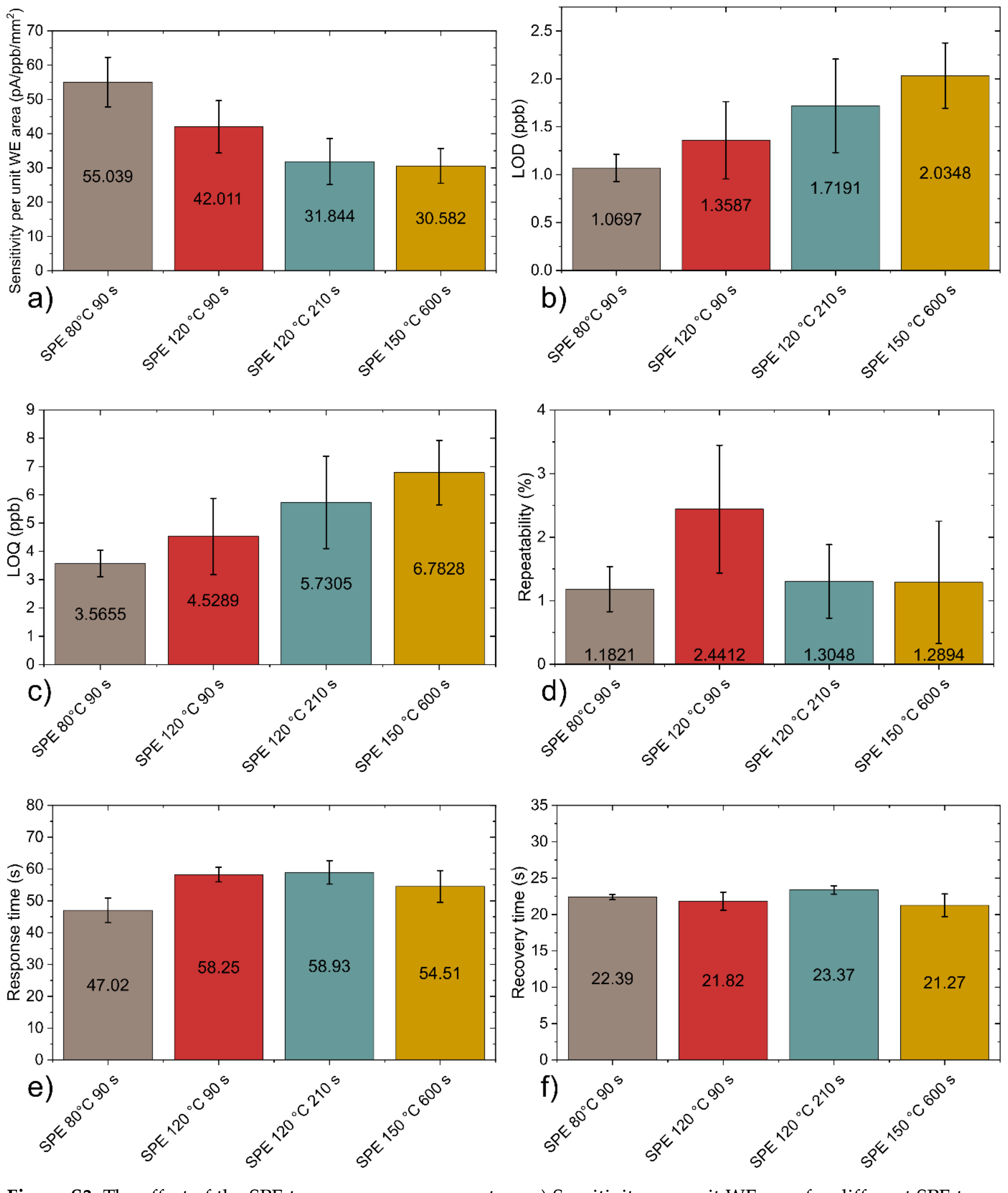

Figure S3. The effect of the SPE type on sensor parameters: a) Sensitivity per unit WE area for different SPE type, b) Limit of detection (LOD) for different SPE type, c) Limit of quantification (LOQ) for different SPE type, d) Repeatability of the sensor response for different SPE type, e) Response time for different SPE type, f) Recovery time for different SPE type; (error bars represents 95% confidence interval).
